# *Begonia catbensis* (sect. Coelocentrum, Begoniaceae), a new species from northern Vietnam

**DOI:** 10.3897/phytokeys.179.65812

**Published:** 2021-06-17

**Authors:** Li-Na Dong, Khang Sinh Nguyen, Yu-Min Shui, Hieu Quang Nguyen, Wei-Bin Xu, Xuan Khu Nguyen

**Affiliations:** 1 Guangxi Key Laboratory of Plant Conservation and Restoration Ecology in Karst Terrain, Guangxi Institute of Botany, Guangxi Zhuang Autonomous Region and Chinese Academy of Sciences, Guilin 541006, Guangxi, China Guangxi Institute of Botany, Guangxi Zhuang Autonomous Region and Chinese Academy of Sciences Guilin China; 2 Institute of Ecology and Biological Resources, Vietnam Academy of Science and Technology, 18 Hoang Quoc Viet Road, Cau Giay, Hanoi, 100000, Vietnam Institute of Ecology and Biological Resources, Vietnam Academy of Science and Technology Hanoi Vietnam; 3 CAS Key Laboratory for Plant Diversity and Biogeography of East Asia, Kunming Institute of Botany, Chinese Academy of Sciences, Kunming 650201, China Kunming Institute of Botany, Chinese Academy of Sciences Kunming China; 4 Center for Plant Conservation of Vietnam (CPC), Vietnam Union of Science and Technology Associations, 25/32 Lane 191, Lac Long Quan Rd., Hanoi, Vietnam Center for Plant Conservation of Vietnam Hanoi Vietnam; 5 Cat Ba National Park, Tran Chau, Cat Hai, Hai Phong, Vietnam Cat Ba National Park Hai Phong Vietnam

**Keywords:** *
Begonia
*, Cat Ba, endangered plant, new species, Vietnam

## Abstract

*Begonia catbensis*, a new species in Begonia sect. Coelocentrum is described and illustrated. The new species was discovered in lowland limestone hills at Cat Ba National Park and can be easily distinguished from all its congeners by having dendritic hairs on the petiole, adaxial veins and stipules, fimbriate bracts and bracteoles, dense conical bullae on the upper surface of the leaf blade, two tepals in the pistillate flowers and a glabrescent ovary with verrucose wings. Based on IUCN Criteria, the species is currently assessed as “Endangered” (D).

## Introduction

*Begonia* L. (Linnaeus 1753) is a highly diverse and widely distributed genus with 2001 currently accepted species (Hughes et al. 2015 onwards). Within the genus, the section Coelocentrum Irmsch. (Irmscher 1939) is mainly distributed in the karst regions and can be easily identified by having 1-locular capsules with parietal placentation (rarely 3-locular with axile placentation) (Ku Chung et al. 2014; Shui et al. 2019). The number of known species in Begonia sect. Coelocentrum has grown from 18 species (Shui et al. 2002) to 73 species (Shui et al. 2019) during the past two decades. The growth of the section has been stimulated by a series of flora diversity surveys conducted by many botanists (Shui and Chen 2005; Fang et al. 2006; Ku et al. 2006; Peng et al. 2007, 2015; Averyanov and Nguyen 2012; Qin et al. 2017; Chen et al. 2018; Radbouchoom et al. 2019; Tong et al. 2019; Liu et al. 2020; Tu et al. 2020). Some species have been merged into sect. Coelocentrum from other sections, for example, *Begonia cavaleriei* H.Lév (Léveillé 1909), *B. pulvinifera* C.-I Peng & Yan Liu (Peng et al. 2006), *B. wangii* Yu (Yu 1948), *B. cylindrica* Liang & Chen (Liang and Chen 1993), *B. leprosa* Hance (Hance 1883) and *B. sinofloribunda* Dorr (Dorr 1999) (Chung et al. 2014; Moonlight et al. 2018; Shui et al. 2019).

In Vietnam, there were only a few publications on *Begonia* before 2000. After describing eight new begonias from Vietnam (Gagnepain 1919), Gagnepain went on to record a total of 18 taxa of the genus for Vietnam in 1921 (Gagnepain 1921). In the late 20^th^ Century, Pham (1991, 1999) made short descriptions in Vietnamese including sketches of 35 species and varieties of native *Begonia* in Vietnam. However, this publication has raised questions for taxonomists because the account does not cite specimens and has some misidentifications. For example, Kiew (2007) excluded four species of *Begonia* out of Pham’s account, renamed one and described three new species for sciences which were misidentified by Gagnepain (1921) and Pham (1991, 1999). The number of species in Begonia sect. Coelocentrum recorded for Vietnam has rapidly increased in recent years, from four species in 2007 (Gagnepain 1921; Nguyen 2004; Shui and Chen 2005; Kiew 2007; Peng et al. 2007) to 21 up to now (Averyanov and Nguyen 2012; Chung et al. 2014; Peng et al. 2014, 2015; Chen et al. 2018; Radbouchoom et al. 2019).

During our field surveys of northern Vietnam in 2019, we found an interesting species of *Begonia* in lowland limestone hills at Cat Ba National Park. This begonia represents characteristics of B. sect. Coelocentrum (Shui et al. 2002; Chung et al. 2014), such as perennial habit, rhizomatous stems, staminate flower with 4 tepals, ovary 1-locular with parietal placentation and 3 unequally winged capsules. In having conical bullae on the upper leaf surface, it is similar to *B. ferox* C.I.Peng & Yan Liu (Peng et al. 2013), *B. fimbribracteata* Y.M.Shui & W.H.Chen (Shui and Chen 2005), *B. masoniana* Irmsch. ex Ziesenhenne (Ziesenhenne 1971), *B. melanobullata* C.I.Peng & C.W.Lin (Peng et al. 2015), *B. montaniformis* C.I.Peng, C.W.Lin & H.Q.Nguyen (Peng et al. 2015), *B. nahangensis* Aver. & H.Q.Nguyen (Averyanov and Nguyen 2012) and *B. variegata* Y.M.Shui & W.H.Chen (Shui and Chen 2005). This plant, however, is obviously differentiated from them by having dendritic trichomes on the petioles, abaxial veins and on the keeled mid-rib of the stipules, 2-tepalled pistillate flowers and glabrescent ovaries with verrucose wings. It is clear that our plant represents a new taxon, therefore we describe and illustrate it here. Furthermore, a key to identify species of B. sect. Coelocentrum with conical bullae on the upper surface of the leaves is provided.

## Materials and methods

Fresh flowers and parts of inflorescences of the new species were fixed and preserved in 50% ethanol for morphological studies. These fixed materials and dried herbarium specimens of the new species are kept at HN and IBK. Herbarium acronyms follow Thiers (2020). Conservation status assessment follows the guidelines in the IUCN Red List Categories and Criteria version 14 (IUCN 2019).

## Taxonomy

### Begonia catbensis

Taxon classificationPlantaeCucurbitalesBegoniaceae

L.N.Dong, K.S.Nguyen & Y.M.Shui sp. nov.

E40FC066-50D2-567A-B8B7-945660A520A7

urn:lsid:ipni.org:names:77217739-1

**Figure 1.**
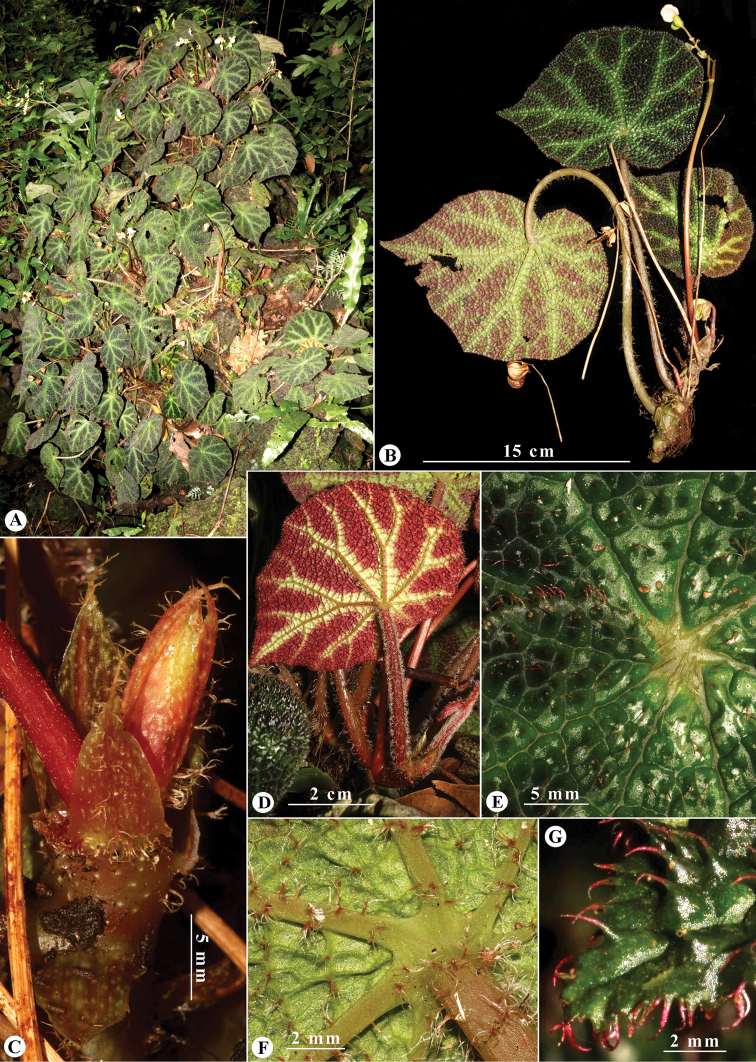
*Begonia catbensis***A** plants growing on rocks in the wild **B** habit **C** apical shoot bearing stipules **D** young leaf, abaxial view **E** part of adaxial leaf surface showing bullae, setae and palmate veins **F** dendritic hairs on petiole and abaxial veins **G** portion of leaf showing reddish setae on bullae and along margin. Photos and layout by K.S. Nguyen & L.N. Dong.

**Figure 2.**
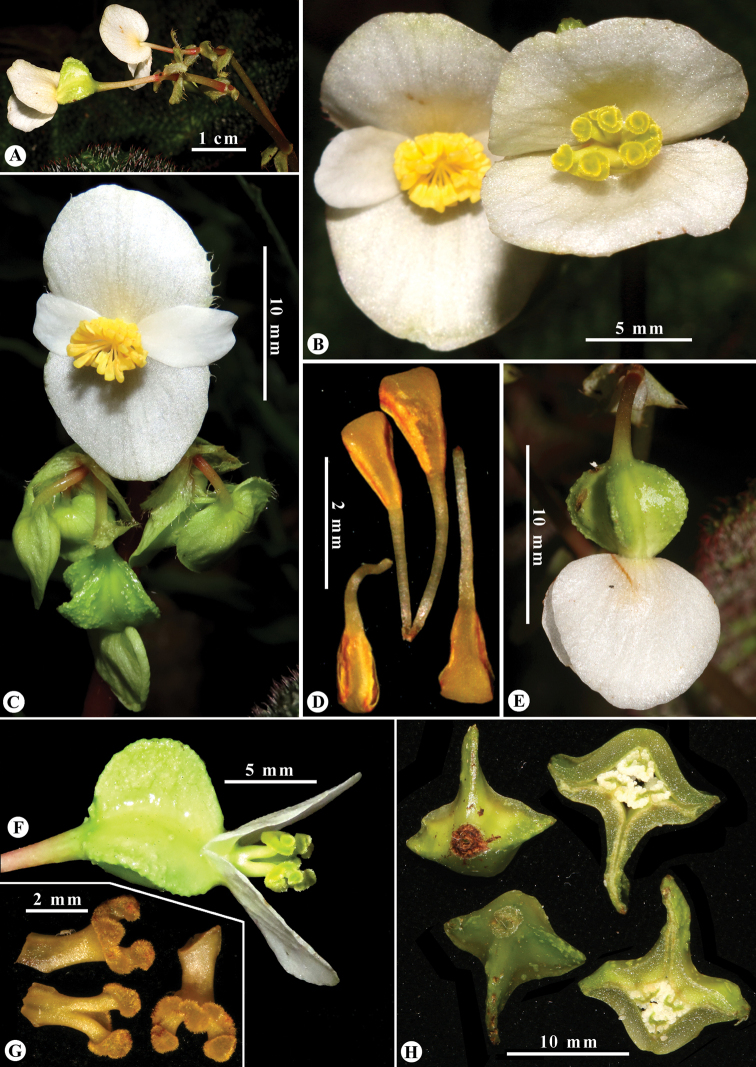
*Begonia catbensis***A** apical part of inflorescence showing peduncle, pedicel, bracts, bracteoles, staminate and pistillate flowers **B** staminate and pistillate flowers **C** staminate flower (upper) in front view **D** stamens **E** pistillate flower-top view **F** pistillate flower, lateral view **G** dissected styles and stigmas **H** fruits and their cross-cut. Photos and layout by K.S. Nguyen & L.N. Dong.

**Figure 3.**
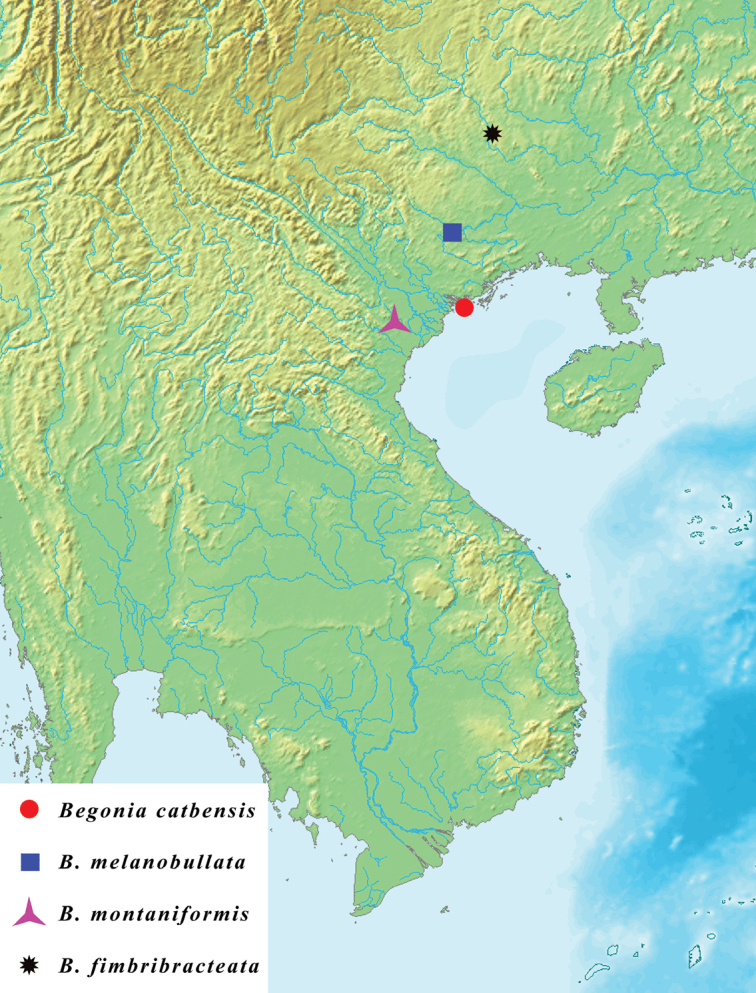
Distribution map of *Begonia catbensis* and its closest related species (The map is modified from https://en.wikipedia.org/wiki/Mainland_Southeast_Asia).

Figs 1 , 2 , 3 , Table 1

#### Diagnosis.

Morphologically similar to several *Begonia* having conically bullate leaves and others with a rugulose leaf surface with white maculation and a ciliolate tepal margin, but can be easily distinguished from them by the dendritic hairs on the petioles, abaxial veins and the keeled mid-rib of the stipules, glabrous peduncles, pistillate flowers with 2 tepals and glabrescent ovaries with verrucose wings.

#### Type.

Vietnam. Hai Phong City, Cat Hai District, Cat Ba National Park, remnants of primary broad-leaved evergreen forest in lowland of limestone hills, around point 20.803333°N, 106.999167°E, 50–70 m a.s.l., flowers white to greenish, fruits green, rare, 24 August 2019, *W.B. Xu*, *K.S. Nguyen*, *C.R. Lin*, *L.N. Dong*, *H.Q. Nguyen* & *X.K. Nguyen 14002* (***Holotype***: HN!; ***Isotypes***: IBK00421271!, HN!).

#### Description.

Herb perennial, monoecious, epipetric, rhizomatous, rooting at nodes, about 25–35 cm tall. Rhizome succulent, elongate, 10–30 cm long, 4–7 mm in diameter, creeping and slightly suberect apically, pale greenish to purplish-green or brownish-red, internodes ca. 1.5 cm long, covered by sparse dendritic hairs. Stipules often persistent, ovate-triangular, herbaceous, brownish-red or purplish when young, later turning pale greenish speckled with purplish patches, 12–16 × 6–12 mm, adaxially glabrous, abaxially keeled, mid-rib with sparse dendritic hairs; margin entire or somewhat undulate and recurved; apex aristate, arista 2–3 mm long. Petiole cylindrical, succulent, 7–16 cm long, 3–4 mm in diameter, densely covered with reddish long-stalked dendritic hairs. Leaves 9–27, basal, alternate, asymmetric, unlobed, broadly ovate, 10–13 × 5–7 cm, papery, adaxially glossy, dark green or rarely brown, with slightly silvery green patches along the veins, surface densely bullate, bullae conical and tipped with a red seta 1.5–2 mm long, abaxially brownish-red to red-brown (maroon), with pale green along major veins, long-stalked reddish dendritic hairs along the veins, base strongly oblique-cordate, margin repand and serrulate with red setae 1.5–2 mm long, apex acuminate; venation palmate with 5–7 primary veins, mid-rib distinct, with 2–4 secondary veins on each side, tertiary veins reticulate or percurrent, minor veins reticulate. Inflorescences axillary, dichasial cymes branched 2–3 times, arising directly from rhizome, pedunculate; peduncle terete, 13–22 cm long, 2.5–3.5 mm thick, glabrous, pale greenish-red to red; bracts and bracteoles not caducous, oblong or oval to ovate, slightly concave at the base and distally bent outwards during flowering, pale green with several longitudinal reddish veins, margin serrate-fimbriate with cilia 1.5–3 mm long, bracts 8–10 × 4–6 mm, slightly larger than bracteoles (6–8 × 2.5–3.5 mm). Staminate flower: pedicel glabrous, 12–19 mm; tepals 4, pure white, outer 2 broadly ovate to suborbicular, 9–14 × 8–12 mm, sparsely puberulent at the proximal margin, inner 2 glabrous, elliptic to oblanceolate, 6–8 × 2.8–3.8 mm; androecium actinomorphic, spherical, 5–6 mm in diameter; stamens 27–32; filaments glabrous, 1.2–1.9 mm long, fused at base, yellowish dull white; anthers somewhat greenish-yellow, narrowly obdeltoid, 1.2–1.5 mm long, widest at apex, 0.8–1 mm wide, apex obtuse, base cuneate, opened by two longitudinal slits with orange margins. Pistillate flowers: pedicel glabrous, 7–14 mm long; tepals 2, suborbicular, 8–10 mm in diameter, greenish-white when young, later turning to dull white or pure white, glabrous, margin entire or slightly undulate with sparsely puberulent at base; ovary green, glabrescent, with verrucose wings, trigonous-ellipsoid, 1-loculed; placentation parietal, with 3 placentae, each 2 branched; styles 3, fused at base or nearly free, glabrous, glossy, yellow, 3–5 mm long, apically C-shaped, stigmatic band twisted. Capsule nodding on a stipe 9–16 mm long, trigonous-ellipsoid, 13–18 mm long, 6–8 mm thick (wings excluded), fleshy, greenish when fresh, 3-winged; wings densely verrucose, unequal, abaxial crescent shaped, 7–9 mm wide, lateral 2.5–3.5 mm wide. Seeds numerous, ellipsoid, brown.

#### Etymology.

The species is named after the type locality, Cat Ba National Park.

#### Phenology.

Flowering in August; fruiting in August – reported earlier.

#### Distribution and habitat.

Cat Ba National Park is composed by many islets. All islets here are limestone mountains. We have had several days to survey in Cat Ba National Park and asked guides working in this National Park for the existence of this species, but we only found and recorded a single population here. Perhaps more populations will be found if more fieldwork is done in the future in this National Park. So far, *Begonia catbensis* is only known from the Cat Ba National Park, Cat Hai District, Hai Phong City, in northern Vietnam. Within its distribution area, the new species grows on semi-shady tops of small rocks and on steep slopes under the broad-leaved evergreen forest in lowlands of limestone hills.

#### Conservation status.

*Begonia catbensis* is considered as a rare species because a single population with less than 200 mature individuals clustered into 10 clumps within an area of ca. 1 km^2^ has been recorded in Cat Ba National Park. Field observation shows that the single population is strictly managed and protected by the staff of the Cat Ba National Park and the number of individuals has been stable for at least two decades. Following the guidelines of the IUCN Red List Categories and Criteria version 14 (IUCN 2019), its conservation status is currently assessed as “Endangered” (D).

#### Taxonomic notes.

Within Begonia sect. Coelocentrum, *B. catbensis* is apparently closest to *B. melanobullata* and *B. montaniformis* from Vietnam (Peng et al. 2015) considering the traits of the stipules and leaves, but strikingly different from them by having dendritic (vs. unbranched) hairs on the keeled mid-rib of the stipules, petioles and abaxial veins of the lamina, 5–7 (vs. 7–9) palmate veins, 2–3-branched inflorescence with 4–10 flowers (vs. 4–6 or 3–8 branched times, with above 20 flowers), glabrous (vs. tomentose or subglabrous) peduncles, glabrous (vs. setulose or velutilous) staminate flower tepals, 2 (vs. 3)-tepalled pistilate flowers and a glabrescent (vs. sessile glandular) ovary. It resembles *B. fimbribracteata* (Shui and Chen 2005) from China in having broadly ovate leaves with adaxial conical bullae on the lamina and serrulate-ciliolate lamina margin, 2–3-branched inflorescence and glabrous bracts with fimbriate margins: however, *B. catbensis* is clearly distinguished by its larger leaves, 10–13 × 5–7 cm (vs. 6–7 × 5–6 cm), acuminate (vs. rotundate) leaf apex and glabrescent (vs. hairy) peduncle, staminate flower tepals and ovary. *Begonia catbensis* is easily distinguishable from *B. nahangensis* (Averyanov and Nguyen 2012) by its papery, asymmetric, broadly ovate leaves (vs. leathery, round to slightly asymmetric broadly oblique-ovate or oblique-reniform leaves) with an acuminate apex (vs. round to rarely broadly obtuse apex) and red setae 1.5–2 mm long along the margin (vs. white soft hairs, 3–5 mm long) and dendritic hairs (vs. villous or woolly villous hairs) on the petiole and abaxial veins and from *B. variegata* (Shui and Chen 2005) in having dark green or rarely brown leaves, with slightly silvery green patches along the veins (vs. dark-purple stripe near the margin of the leaves and dark brown wide bands along main veins), dendritic hairy petioles (vs. hirsute-villous), glabrous (vs. glandular hairy) peduncles, outer tepals and ovary and greenish-white to white (vs. greenish or greenish-yellow) flowers. A detailed comparison of the new species with its most morphologically similar species is listed in Table 1. To help quickly identify *Begonia catbensis* from its congers, a key to Begonia sect. Coelocentrum with conical bullae on their leaves is provided.

**Table 1.** Morphological comparison of *Begonia catbensis*, *B. melanobullata*, *B. montaniformis* (Peng et al. 2015) and *B. fimbribracteata* (Shui and Chen 2005).

Characters	*B. catbensis*	*B. melanobullata*	*B. montaniformis*	*B. fimbribracteata*
Stipule	glabrous, except for the keeled midrib with sparse dendritic hairs	glabrous, except for the keeled midrib with unbranched hairs	glabrous, except for the keeled midrib with unbranched hairs	subglabrous
Petiole	densely covered by reddish dendritic trichomes	densely white villous when young, brownish tomentose or subglabrous later	densely white villous	sparse strigae 1–2 mm long
Leaf color	adaxially dark green or rarely brown, with slightly silvery green patches along veins; abaxially brownish red to red-brown (maroon)	adaxially emerald green to yellowish green; abaxially pale green, reddish on veins and bullae	adaxially blackish-malachite green, purplish-olive or dark bluish-brown, with silvery green zone along main veins; abaxially pale green, reddish	adaxially green or brown, with white dots along major veins; abaxially reddish
Leaf bulla	tipped by a reddish seta 1.5–2 mm long	tipped by a velutinous hair 6–10 mm long	tipped by 2–6 peak-like hispidulous protrusions	tipped by a seta 1.5–2 mm long
Leaf margin	repand, serrulate and ciliolate	repand villous when juvenile	repand to shallowly denticulate and ciliate	serrulate and ciliate
Leaf apex	acuminate	caudate	acute to acuminate	rounded
Venation	5–7-veined palmate	7–9-veined palmate	7–9-veined palmate	6–7-veined palmate
Vein on abaxial surface	covered by reddish dendritic hairs	brownish tomentose	densely brownish-white tomentose	covered by strigae 1–1.5 mm long
Inflorescence	branched 2–3 times, 4–10 flowers	branched 4–6 times, numerous flowers (>20)	branched 3–8 times, numerous flowers, up to above 30	branched 2–3, ca. 5 flowers
Peduncle	glabrous	tomentose	tomentose to subglabrous	sparsely hairy
Bract	glabrous, margin serrate-fimbriate with cilia 1.5–3 mm long	glabrous, margin tomentose	abaxially velutinous along midrib, margin tomentose	glabrous, margin serrulate-fimbriate with cilia 1–2 mm long
Staminate flower tepal	abaxially glabrous	abaxially sparsely setulose	abaxially sparsely velutinous	abaxially sparsely pilose
Pistillate flower tepal number	2	3	3	3
Tepal margin	sparsely ciliolate below middle	entire, not hairy	entire, not hair	entire, not hairy
Tepal color	greenish white to white on both surface when opened	adaxially yellowish-pinkinsh, abaxially reddish	adaxially yellowish-greenish, abaxially reddish-green	pink or white
Ovary	glabrescent	sparsely dotted with sessile glands	sparsely sessile-glandular	sparsely hairy

Amongst Begonia sect. Coelocentrum with a rugulose leaf surface, *Begonia catbensis* somewhat resembles *B. ningmingensis* D.Fang, Y.G.Wei & C.I.Peng and *B. retinervia* D.Fang, D.H.Qin & C.I.Peng from China (Fang et al. 2006) in leaf shape, with white maculation of the adaxial leaf surface and tepals with ciliolate margins, but it is strikingly differentiated from them in having a conically bullate (vs. rugulose) leaf surface, adaxially glabrous (vs. subsessile glandular) stipules with dendritic hairs on the mid-rib (vs. villous or glabrous), dendritic (vs. villous) petioles and abaxial veins, glabrous (vs. sparsely minute subsessile glandular) abaxial surface of staminate flower tepals and ovary and 2 (vs. 3) tepals of pistillate flower.

### Identification key to *Begonia* species with conical bullae on leaf surface within sect. Coelocentrum

**Table:** 

1	Dendritic hairs on petiole, along adaxial palmate veins and keeled mid-rib of stipule	***B. catbensis***
–	Hairless or with unbranched hairs on petiole, along adaxial palmate veins and keeled mid-rib of stipule	**2**
2	Leaf blade with brown or dark purple maculation on adaxial surface; exterior surface of tepal with glandular hairs	**3**
–	Leaf blade without obvious maculation on adaxial surface; exterior surface of tepal without glandular hairs	**4**
3	Leaf abaxially densely villous and tomentose, adaxially having a dark purple ring near the margin; peduncles and pedicels with dense glandular strigae	***B. variegata***
–	Leaf abaxially sparsely long strigose, without a dark purple ring near the margin; peduncles and pedicels subglabrous	***B. masoniana***
4	Bullae 2–4 tipped	***B. montaniformis***
–	Bullae with a single tip	**5**
5	Leaf apex obtuse or rounded	**6**
–	Leaf apex acute to acuminate or shortly caudate	**7**
6	Abaxial veins densely white woolly-villous; peduncle glabrous; bract margin entire	***B. nahangensis***
–	Abaxial veins laxly strigose; peduncle hairy; bract margin fimbriate	***B. fimbribracteata***
7	Dense conical bullae present on all leaves; hairs on bullae tip persistent; inflorescence branched 4–6 times; male flowers greenish; ovary sparsely dotted with sessile glands	***B. melanobullata***
–	Conical bullae sparsely present or absent on immature leaves; hairs on bullae tip deciduous; inflorescence branched 3–4 times; male flowers pale pinkish-yellow; ovary glabrous	***B. ferox***

## Supplementary Material

XML Treatment for Begonia catbensis
